# Validation of Brazilian Version of the Sexual Desire Inventory 2 (SDI-2)

**DOI:** 10.61622/rbgo/2024rbgo78

**Published:** 2024-10-23

**Authors:** Denisse Cartagena-Ramos, Miguel Fuentealba-Torres, Flávio Rebustini, Josilene Alves, Alessandro Scholze, Lúcia Alves da Silva Lara, Ricardo Arcêncio, Lucila Castanheira Nascimento

**Affiliations:** 1 Universidad Andrés Bello Facultad de Enfermería Santiago Chile Facultad de Enfermería, Universidad Andrés Bello, Santiago, Chile.; 2 Universidad de los Andes Santiago Chile Universidad de los Andes, Santiago, Chile.; 3 Universidade de São Paulo Escola de Artes, Ciências e Humanidades São Paulo SP Brazil Escola de Artes, Ciências e Humanidades, Universidade de São Paulo, São Paulo, SP, Brazil.; 4 Universidade Federal do Mato Grosso Cuiabá MT Brazil Universidade Federal do Mato Grosso, Cuiabá, MT, Brazil.; 5 Universidade Estadual do Norte de Paraná Jacarezinho PR Brazil Universidade Estadual do Norte de Paraná, Jacarezinho, PR, Brazil.; 6 Universidade de São Paulo Faculty of Medicine of Ribeirão Preto Ribeirão Preto SP Brazil Faculty of Medicine of Ribeirão Preto, Universidade de São Paulo, Ribeirão Preto, SP, Brazil.; 7 Universidade de São Paulo Escola de Enfermagem São Paulo SP Brazil Escola de Enfermagem, Universidade de São Paulo, São Paulo, SP, Brazil.; 8 Universidade de São Paulo Escola de Enfermagem de Ribeirão Preto Ribeirão Preto SP Brazil Escola de Enfermagem de Ribeirão Preto, Universidade de São Paulo, Ribeirão Preto, SP, Brazil.

**Keywords:** Libido, Sexual desire, Cross-cultural comparison, Psychometrics, Sexual behavior, Surveys and questionnaires

## Abstract

**Objective:**

To traslate and validate of the Brazilian version of the SDI-2.

**Methods:**

This was a cross-sectional study. The cultural adaptation considered the stages of initial translation, synthesis of translations, evaluation by a committee of experts from different regions of Brazil, back-translation, and pre-test. The content validity and psychometric proprieties was assessed.

**Results:**

Ten specialists participated in the cultural adaptation of the SDI-2. The content validity showed a Content Validity Ratio (CVR) ≥ 0.75 (*p* = 0.05). A total of 674 subjects participated in the field study. The Exploratory Factorial Analysis (EFA) presented factor loads ≥ 0.445, and commonalities ≥ 0.40; and two dimensions represented 77% of the total variance explained. The Confirmatory Factorial Analysis CFA presented *X*^2^/df = 4.265; the Root Mean Square Error of Approximation RMSEA = 0.110; the Non-Normed Fit Index NNFI = 0.946; the Comparative Fit Index (CFI) = 0.963; the Goodness of Fit Index GFI = 0.986; and the Adjusted Goodness of Fit Index AGFI = 0.979 for a two-factor model. The coefficient values for the total SDI-2 score were 0.91 for Cronbach's alpha, 0.91 for McDonald's Omega, and 0.97 for the Greatest Lower Bound GLB coefficients. The invariance between sexes was 0.01 for the ΔCFI and ΔRMSEA, showing model stability for these two populations.

**Conclusion:**

The Brazilian version of the SDI-2 is self-report, valid, reliable and invariant across sex.

## Introduction

Sexual desire is an early component in the human sexual response^(1)^ characterized by emotional, biological, and cognitive aspects that motivate the search for sexual intimacy.^(2)^

Hypoactive Sexual Desire Disorder (HSDD) is the main sexual dysfunction in women^(3)^ and the second most common in men.^(4,5)^ Global epidemiological studies estimate that the prevalence of HSDD affects between 17% and 50% of the female population^(6,7)^ and 14% to 17% of the male population.^(6)^ In Brazil, an epidemiological study indicated that HSDD is the most relevant sexual dysfunction in women, affecting 26.7% of them and 12.3% of men.^(8)^ However, HSDD indicators in the Brazilian population were not estimated by the use of measuring instruments for sexual desire. Therefore, it is currently difficult to estimate the magnitude of sexual desire problems in this population due to the lack of validated instruments and methodological differences in the available studies.^(9)^ Thus, the Fourth International Consultation on Sexual Medicine^(10)^ expressed the need for validated and reliable measurement instruments to estimate the real magnitude of sexual problems in the populations.

There are no validated instruments that assess sexual desire in the Brazilian population, which hinders the development of epidemiological studies and contributes to the estimation of the uncertain prevalence of HSDD cases.

A systematic review identified and analyzed the methodological quality and levels of evidence of validated sexual desire instruments.^(11)^ In this review, the Sexual Desire Inventory 2 (SDI-2)^(12)^ was highlighted as an inventory capable of measuring sexual desire in men and women through a single instrument based on its adequate psychometric properties. This is a self-report type inventory and has been validated in Germany,^(13)^ Spain,^(14)^ and Portugal^(15)^ with use in different social and cultural contexts of respondents.^(16,17)^

Due to the urgent need to have an instrument to measure sexual desire of men and women in Brazil, the present study was conducted to translate and validate of the Brazilian version of the Sexual Desire Inventory 2 (SDI-2).

## Methods

This was a cross-sectional study developed between December 2017 and October 2018 with Brazilian men and women in order to obtain evidence of validation of the Brazilian version SDI-2 for content and construct. The SDI-2 includes 14 items; four of them with scores ranging from 0 to 7 and related to frequency of desire; the remaining 10 items with scores ranging 0 to 8. The items from 1 to 8 are added to dyadic sexual desire and the items 9 to 11 are added to solitary sexual desire.^(12)^ In addition, there is an online version of the Sexual Desire Inventory 2 (SDI-2) which has shown evidence of robust validity in the Brazilian online version of the Sexual Desire Inventory 2^(18)^ . A team of two translators and two retro translators English/Brazilian Portuguese bilingual, a committee of ten experts/lay people participated in the cultural adaptation stage. Adult men and women participated in the pre-test over 18 years of age, in a stable marital relationship for a period of six months or more, and with the ability to read, write, and understand the content of the SDI-2 instrument.

### I. The validity of content: cultural adaptation of the Brazilian version of the SDI-2

**Figure 1:**
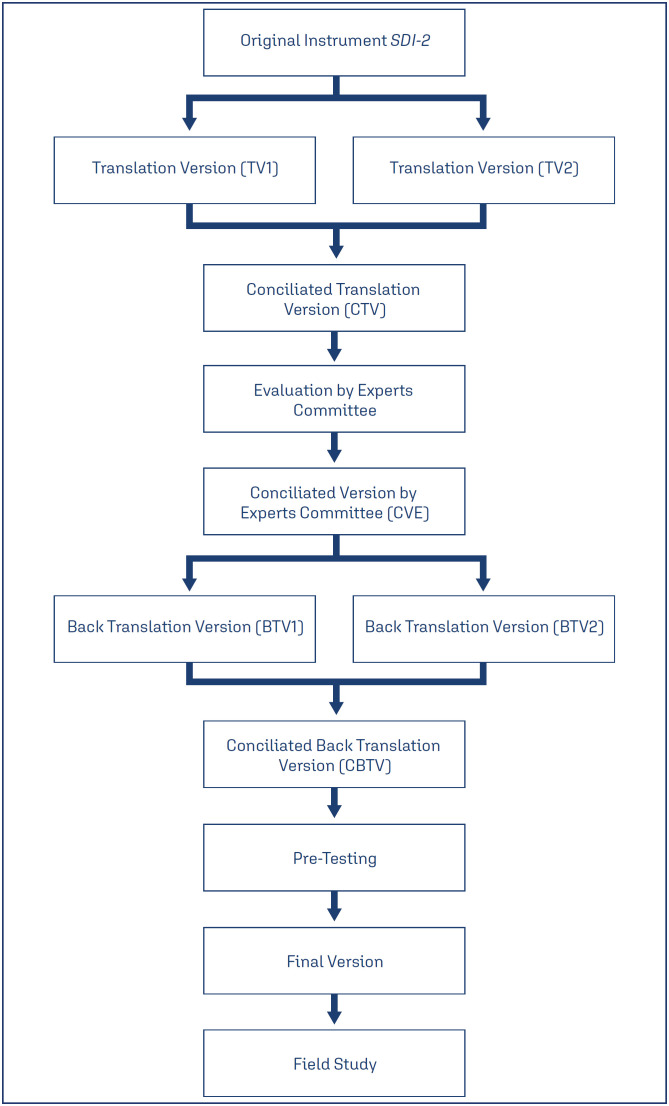
Flow Diagram to the Validation of *Sexual Desire Inventory 2*

Source: Flow diagram to the Validation of Sexual Desire Inventory 2 based on the stages of Beaton et al. (2000)^(18)^ * Modification in the order of the back-translation stage and the committee of experts according to Ferrer et al. (1996).^(19)^

Five stages were developed in the cultural adaptation of the SDI-2: initial translation, synthesis of translations, evaluation by the expert committee, retro translation, and field pre-test.^(19,20)^ The stage of evaluation by the expert committee was carried out prior to the back-translation stage. This modification was adopted based on previous studies^(21,22)^ demonstrating the optimization of time and resources in the process. Figure 1 shows the flowchart with the stages developed in the process of instrument cultural adaptation.

- Step 1 - Initial Translation (T1/T2): The original version of the SDI-2 was independently translated into Brazilian Portuguese by two English/Brazilian Portuguese translators (T1/T2) generating two translated versions (TV1/TV2). Both translated versions were evaluated by a team of eight public health researchers, including six authors of the present study and two external researchers, who compared the translated versions with the objective of identifying similarities and differences and facilitating analysis in the next step.
- Step 2 - Synthesis of translations: The main author of this study and her adviser, both with knowledge of the English and Brazilian Portuguese languages, analyzed the two TV2/TV2 translated versions to generate a reconciled translated version (RTV).
- Step 3 - Review by the expert committee: The reconciled translated version (RTV) was forwarded to the committee of experts. This committee should have at least two reviewers who are experts in the content area and, at least one expert who is expert in instrument construction, in addition, the inclusion of lay people in the expert panel.^(23)^ This committee was comprised of seven health professionals, and the lay people were represented by three individuals from the community. The validity of the RTV content was assessed qualitatively and quantitatively by the committee of experts.^(23,24)^ Equivalences were analyzed in the qualitative evaluation. The conceptual equivalence by items and semantics aimed to evaluate the relevance of dimensions, relevance of items, and equivalence of meanings, respectively.^(25)^ The quantitative evaluation analyzed the content through the Content Validity Ratio (CVR) adopting values ≥0.75 (*p* = 0.05) as acceptable.^(24)^ The suggestions of the committee of experts were considered by the researchers with minimum adjustments. The version originated in this process was identified as RTV.
- Step 4 Retro-translation: To optimize time and resources, the RTV was retro-translated by two English/Brazilian Portuguese translators generating two back-translated versions (BT1 and BT2). Both versions were compared by the lead author and her advisor, and the reconciled retro-translated version was generated (RBT). This version was sent to the author of the original SDI-2 instrument for her evaluation, provision of suggestions, and agreement considering the RBT original version in order for this study to proceed.
- Step 5 Pre-test: Upon the agreement from the author of the original SDI-2 instrument to advance in the process of validation of the instrument in our study, the sample for the pre-test stage of the RBT was calculated following the recommendations of 30 to 40 participants.^(26)^ The content of 14 items was evaluated based on the concordance index from the participants’ responses. Concordance was evaluated using a form composed of three options: 1 = "I understand," 2 = I am undecided," and 3 = "I do not understand."

### II. Construct validity: a field study

The criterion of > 20 participants per item was used for sample calculation.^(27)^ The data were collected by a trained team of collectors composed of five researchers. The collection was carried out in parks, bus terminals, and neighborhoods of cities located in the Central-Western, Southeastern, and Southern regions of Brazil. The construct validation was performed by applying Exploratory and Confirmatory Factor Analysis.

The adequacy of the correlation matrix was assessed using the Bartlett Sphericity test and the Kaiser-Meyer-Olkin test (KMO).

Exploratory analysis was based on the correlation considering the amplitude of the scale from 0 to 8.^(28,29)^ The parallel analysis was applied polychoric through the Optimal implementation of Parallel Analysis (PAN) for dimensionality testing.^(30)^ UNICo (One-dimensional Congruence) > 0.95, ECV (Explained Common Variance) > 0.85, or MIREAL (Mean of Item Residual Absolute Loading) < 0.30^(31)^ were used as complementary forms of testing the unidimensionality or multidimensionality of the model.

The Robust Unweighted Least Squares (RULS) associated with a bootstrap (*n* = 5000) with Direct Oblimin rotation were used for data extraction.

The factorial structure of the two factors was initially evaluated according to the original model of the instrument. The factorial solution was evaluated through saturation of the factorial loads > 0.40, total variance explained > 60%, and commonalities > 0.40.^(27)^ Pratt's Importance Measurements was used as a way of complementing the factorial solution.

Five factor fit indices were used for the Confirmatory Factor Analysis (CFA): Root Mean Square Error of Approximation (RMSEA) ≤ 0.08, Non-Normed Fit Index (NNFI; Tucker & Lewis) > 0.95, Comparative Fit Index (CFI) > 0.95, Goodness of Fit Index (GFI) > 0.95, and Adjusted Goodness of Fit Index (AGFI) > 0.95.^(32)^

Reliability was assessed by Cronbach's alpha,^(33)^ Greatest Lower Bound (GLB),^(34)^ and McDonald's Omega^(35)^ coefficients.

The quality of the factorial solution and replicability of the model were tested by the Generalized H (GH) Index, and the quality and effectiveness of the factor score estimates were calculated by the Factor Determinacy Index (FDI) and the ORION marginal reliability index.^(31)^

The metric invariance was tested by the ΔCFI and ΔRMSEA between male and female samples. The difference between the models should not be greater than 0.01 for ΔCFI and 0.015 for ΔRMSEA.^(36)^

The collected data were inserted into a database and validated by double typing in a Microsoft Excel spreadsheet. Statistical analyses were performed using the FACTOR software version 10.8.04 and the IBM SPSS AMOS software version 22.0. The descriptive statistics of the sociodemographic variables were performed, and minimum and maximum frequencies and percentages were calculated. Measurements of central tendency and dispersion were calculated for the variable age.

This research was approved by the University Institutional Review Board (Process nº 281/2017 of July 06, 2017). Approved by the Ethics Committee of the University of São Paulo under process number (CAAE no 79325517.2.0000.5393). All participants signed the free and informed consent form according to Resolution 466/12 of the Brazilian National Council of Health, which guides research developed with human beings. All research procedures complied with the 1964 Helsinki Declaration and its later amendments or ethical standards.

## Results

### Cultural adaptation

A multidisciplinary team of professionals and people from the community participated in the cultural adaptation of the instrument. Two translators and two retro-translators participated in the translation and back-translation stages. Seven professionals and three lay people from different regions of Brazil participated in the committee of experts. This committee was comprised of seven health professionals, including a psychometrist from the Central-Western region of Brazil; a sexologist from the Southeastern region; two psychologists, one from the Northeastern region and one from the Southern; a urologist from the Southeastern region; a gynecologist from the Southeastern region; and a nurse, a specialist in sexuality, from the Midwestern region. The lay people were represented by a 49-year-old woman, a cleaning assistant from the southern region of Brazil; a 45-year-old man, self-employed in the Southeast region of Brazil; and a 68-year-old woman, manager of a company in the Southeast region of Brazil. In the pre-test, 36 participants were invited, of whom 16 were males and 16 females.

- Step 1: Initial Translation (T1/T2): The evaluation of the two translated versions (TV1/TV2) resulted in modifications suggested by the research team. The original phrase "This questionnaire reports on your level of sexual desire" was modified to "This questionnaire asks about your level of sexual desire." In items 1, 2, 3, 7, 8, and 9, the word "female partner" was replaced by "partner" to include all options for gender orientation.
- Step 2: Synthesis of translations: The CTV was developed after consensus was reached over the following discrepancies. In item 10, the phrase "Would you like to practice sexual behavior alone?" was replaced by "Would you have liked to sexually satisfy yourself alone?" In item 14, the word "activities" was replaced by "any activity," and the phrase "You could, comfortably, be without any type of sexual activity" was replaced by "You would comfortably be without any type of sexual activity."
- Step 3: Review by the Expert Committee: The authors adapted the words that were identified as difficult to understand in the qualitative evaluation of the expert committee. Subsequently, the quantitative evaluation showed values of CVR ≥ 0.75 (*p* = 0.05), except for items 4, 5, 6, and 13. These items obtained values below the critical CVR value because the specialists considered them not essential; but they were not withdrawn because the committee still considered them useful. Therefore, we opted to test the evidence of the validation of these items in the construct stage. Moreover, the exclusion of these items in this phase would cause a significant change in the original instrument before being tested in the field, making it impossible to more accurately analyze their functionality in another phase of the validation process.
- Step 4: Back-translation: The author of the original SDI-2 suggested replacing the CBTV phrases "will not be identified" with "will be maintained in privacy." In item 3, the phrase "How strong is your desire to have sex with a partner?" was replaced with "How strong is your desire to get involved in sexual activity with a partner?"
- Step 5: Pre-test: The agreement index was ≥ 91.7%.

### Field study

A total of 674 participants were recruited; 13 participants were excluded due to incomplete filling of data collection instruments. The final sample contained 661 participants. A percentage of 65.6% (n = 431) of participants were women and 35.4% (n = 230) were men. Table 1 shows the sociodemographic characteristics of the study participants.

**Table 1:** Descriptive characteristics of the respondents

Characteristics		Min* – Max**	Mean ± SD Median
Age in years		18 - 78	29.78 ± 11.1 27.0
		n(%)	
Region of Brazil			
	North	4(0.6)	
	Northeast	2(0.3)	
	Mideast	237(35.2)	
	Southeast	201(29.8)	
	South	230(34.1)	
Civil status			
	Married / Unemployed	249(36.9)	
	Separated / Divorced	22(3.3)	
	Not married	394(58.5)	
	Widower	8(1.2)	
Sex			
	Female	444 (65.9)	
	Male	230 (34.1)	
Education			
	Complete primary education	20(3.0)	
	Incomplete elementary school	41(6.1)	
	Complete high school	154(22.8)	
	Incomplete high school	45(6.7)	
	Complete Higher Education	158(23.4)	
	Incomplete higher education	256(38.0)	
Occupation			
	Retired / Pensioner	16(2.4)	
	Unemployed	51(7.6)	
	From home	43(6.4)	
	Employee	314(46.6)	
	Student	235(34.9)	
	Missing	4(0.6)	
Race			
	Yellow	12(1.8)	
	White	333(49.4)	
	Indigenous	10(1.5)	
	Brown	226(33.5)	
	Black	69(10.2)	
Chronic disease			
	No	568(84.3)	
	Yes	90(13.4)	
Religion (Active participation)			
	No	568(84.3)	
	Yes	90(13.4)	
Relationship			
	No	420(62.3)	
	Yes	237(35.2)	
	Missing	5(2.5)	
Sexual Preference			
	By women	402(59.6)	
	By the men	199(29.5)	
	For men and women	38(5.6)	
	Rather not answer	14(2.1)	
	Missing	9(3.2)	
Sexual activity			
	Two or three times a month	110(16.3)	
	Twice a week	107(15.9)	
	More than once a day	15(2.2)	
	Not once	91(13.5)	
	Three or four times a week	148(22.0)	
	Once a month	66(9.8)	
	Once a day	37(5.5)	
	Once a week	85(12.6)	

* Minimum;

** Maximum; SD – Standard deviation

### Construct validity

The adequacy of the sample presented KMO = 0.88 and Bartlett's Sphericity test of 73.1 (*p* < 0.010) as significant and indicative of good data factorability. The analysis of dimensionality performed by the robust parallel analysis confirmed the existence of two dimensions. The complementary indicators for dimensionality reaffirmed a multidimensional model with UNICO = 0.913; *ECV =* 0.719, and *MIREAL =* 0.378. The two-dimensional factorial solution represented 77% of the total variance explained for the two-dimensional model. The configuration was defined as Factor 1, "sexual desire in the relationship," retaining items 1, 2, 3, 4, 5, 6, 7, 8, and 9; and Factor 2, related to "solitary sexual desire," retaining items 10, 11, 12, and 13. Table 2 presents the values of factor loads, commonalities, and Pratt's Measurements. Table 3 shows the values of adjustment indexes observed in the two-factor model of the confirmatory factor analysis (CFA). The factorial loads of the model showed values ≥ 0.445. The commonalities presented values ≥ 0.40. Pratt's Measurements technique reaffirmed the alignment of the items in two factors, corroborating the solution proposed in the factorial analysis.

**Table 2:** Standardized factor loadings, communalities (h^2^), Pratt's Measure and confirmed of factorial solution from the Exploratory Factorial Analysis

Sexual Desire Inventory 2 (SDI-2)	Inventário do Desejo Sexual 2 (IDS-2)	Factor loading		Communalities	Pratt´s Measure	
		F1	F2	h^2^	F1	F2
1. During the past month, how many times would you like to have engaged in sexual intercourse with one partner (for example, touching one another's genitals, giving or receiving oral stimulation, having sex, etc.)?	1. Durante o último mês, quantas vezes você gostaria de ter se envolvido em relações sexuais com um(a) parceiro(a) (por exemplo, tocando os genitais um do outro, dando ou recebendo estimulação oral, tendo relações sexuais, etc.)?	0.62	0.09	0.44	0.41	
2. During the past month, how many times have you had sexual thoughts involving a partner?	2. Durante o último mês, quantas vezes você teve pensamentos sexuais envolvendo um(a) parceiro(a)?	0.61	0.12	0.44	0.40	
3. When you have sexual thoughts, how strong is your desire to engage in sexual activity with a partner?	3. Quando você tem pensamentos sexuais, quão forte é seu desejo de se envolver em atividade sexual com um(a) parceiro(a)?	0.86	-0.02	0.73	0.73	
4. When you see an attractive person for the first time, how strong is your sexual desire?	4. Quando você vê uma pessoa atraente pela primeira vez, quão forte é seu desejo sexual?	0.48	0.33	0.46	0.29	
5. When you spend time with an attractive person (for example, at work or school), how strong is your sexual desire?	5. Quando você passa tempo em companhia de uma pessoa atraente (por exemplo, no trabalho ou na escola), quão forte é seu desejo sexual?	0.44	0.31	0.40	0.25	
6. When you are in a romantic situation (for example, at a candlelit dinner, walking on the beach, etc.), how strong is your sexual desire?	6. Quando você está em uma situação romântica (por exemplo, em um jantar à luz de velas, caminhando na praia, etc.), quão forte é seu desejo sexual?	0.73	-0.04	0.51	0.51	
7. How strong is your desire to engage in sexual activity with a partner?	7. Quão forte é seu desejo de se envolver em atividade sexual com um(a) parceiro(a)?	0.89	-0.03	0.77	0.77	
8. How important is it for you to satisfy your sexual desire through any activity with a partner?	8. Quão importante é para você satisfazer seu desejo sexual através de qualquer atividade com um(a) parceiro(a)?	0.74	-0.07	0.52	0.52	
9. Compared to other people of your age and gender, how do you assess your desire to behave sexually with a partner?	9. Comparado com outras pessoas da sua idade e sexo, como você avalia seu desejo de se comportar sexualmente com um(a) parceiro(a)?	0.77	-0.00	0.59	0.59	
10. During the past month, how often would you like to have sexually satisfied yourself (eg masturbating, touching your genitals, etc.)?	10. Durante o último mês, quantas vezes você gostaria de ter se satisfeito sexualmente sozinho (por exemplo, se masturbando, tocando seus genitais, etc.)?	0.07	0.79	0.68		0.66
11. How strong is your desire to engage in sexual activity alone?	11. Quão forte é seu desejo de se envolver em atividade sexual sozinho?	-0.00	0.94	0.88		0.88
12. How important is it for you to satisfy your sexual desires alone?	12. Quão importante é para você satisfazer seus desejos sexuais sozinho?	-0.05	0.98	0.93		0.93
13. Compared to other people of your age and sex, how do you assess your desire to satisfy sexually alone?	13. Comparado a outras pessoas da sua idade e sexo, como você avalia seu desejo de se satisfazer sexualmente sozinho?	0.00	0.89	0.81		0.81

F1 - Dyadic sexual desire; F2 - Solitary sexual desire; h2 - Communalities; Pratt's - Pratt's importance measures; p˂0.05

**Table 3:** Summary of Goodness of Fit Statistics for *Sexual Desire Inventory 2 (SDI-2)*

Model	X^2^	df	X^2^/df	RSMEA	NNFI	CFI	GFI	AGFI
Two Factors	226.616	53	4.265	0.110	0.946	0.963	0.986	0.979

AGFI - Adjusted Goodness of Fit Index; CFI - Comparative fit index; df - difference test; GFI - Goodness-of-fit index; NNFI - Non-Normed Fit Index; RMSEA - Root mean square error of approximation

### Reliability, quality, and replicability of the factorial solution

The Cronbach's alpha coefficient was 0.91 for the instrument, 0.88 for the subscale "desire in the relationship," and 0.92 for "solitary desire." The other two reliability techniques also showed satisfactory results: The McDonald's Omega and the Greatest Lower Bound (GLB) coefficients were 0.91 and 0.97, respectively. The stability of the Brazilian version of the SDI-2 was evaluated through the Generalized H (GH) Index, presenting the value of 0.92 for the subscale of "solitary desire" and 0.96 for the subscale of "sexual desire in the relationship." The quality and effectiveness of the estimates were evaluated through the Factor Determinacy Index, which presented values of 0.96 and 0.98 for the first and second factors, respectively. The ORION marginal reliability index indicated values of 0.92 and 0.96 for the first and second factors, respectively. All indicators were above the stipulated minimum limits ^(27^^).^

### Invariance

The metric invariance (Table 4) showed stability between the models for female and male sexes. The ΔCFI and ΔRMSEA resulted in 0.01 and within the limit established in the literature ^(36^^).^

**Table 4:** Summary of Goodness of Fit Statistics for *Sexual Desire Inventory 2 (SDI-2)* Metric Invariance Across Sex

	Model	n	X^2^	df	X^2^/df	CFI	RMSEA	ΔCFI	ΔRMSEA
Female	Two factor	431	160.892	53	3.037	0.966	0.103	0.011	0.011
Male	Two factor	230	106.041	53	3.019	0.955	0.114		

X2 - Chi Square df - degrees of freedom; CFI - Comparative fit index; ΔCFI – Comparative fit index delta; ΔRMSEA – Root mean square error approximation delta

## Discussion

The present study aimed to translate and validate of the Brazilian version of the SDI-2. The initial translation, as the first stage of cultural adaptation, was essential to adapt the instrument and improve the understanding and interpretation of concepts in the original instrument.^(19)^ Finally, the study evaluated the content of the Brazilian version of the SDI-2 according to the recommendations to demonstrate content validity of measuring instruments.^(23,24)^

The sample size allowed for great precision of the adopted techniques used in the construct validation. One study showed that more than 20 participants per item could significantly reduce errors and inaccuracies in the solutions of psychometric models—such as the average of items incorrectly classified in each factor, the mean of the error in factor loads, and the percentages of *Heywood* cases.^(37)^ In the SDI-2 validation studies performed in other cultures, the following sampling criteria were used in other versions: the Canadian,^(12)^ Spanish,^(14)^ and Portuguese^(15)^ versions used ≥ 20 participants per item; however, the German version^(13)^ used ≥ 10 participants per item.

The use of the Optimal implementation of Parallel Analysis (PA) to evaluate the instrument's design allowed to ensure the factorial structure of the SDI-2. Several studies have recommended the use of this technique because it is a robust and precise technique for this purpose.^(38,39)^ The original Canadian^(12)^ and German ^(13)^ versions evaluated the design through the eigenvalue; the Spanish^(14)^ Portuguese^(15)^ versions did not describe the techniques used to evaluate the SDI-2 design.

The use of the Robust Unweighted Least Squares (RULS) in this article aimed to reduce residues from the matrices.^(40)^ Both original Canadian^(12)^ and Portuguese^(15)^ versions extracted the factors through the Maximum Likelihood Factor Analysis (MLFA); while the German version used the Components Principals Analysis (CPA), and the Spanish version^(14)^ did not report the technique used to extract factors.

The two-factor model carried the same items proposed in the original version of the instrument.^(12)^ These results coincide with the results reported by the validation study of the German version of this instrument,^(13)^ although the results differ from the results reported in the Spanish^(14)^ and Portuguese^(15)^ versions. The divergences are possibly related to the different techniques applied, the quality of the data used, and the sample size, which directly affects the values of factorial loads, commonalities, and other AFE indicators.^(37)^

Sexual desire has a multidimensional characteristic and is therefore associated with subjective, social, environmental, cultural, and relational aspects. Such dimensions are not always accessible by psychometric instruments, which can lead to different results in validation studies developed in different cultures. The AFC presented satisfactory levels of adjustment indexes of the model, corroborating the model established by the exploratory analysis.

The study showed that the Brazilian version of the SDI-2 presented satisfactory results, with a Cronbach's alpha coefficient of 0.91 and 0.88 for the subscales of sexual desire in the relationship and solitary sexual desire, respectively. These findings coincide with the original SDI-2 study, which obtained values of the Cronbach's alpha coefficient of 0.86 and 0.96, respectively.^(12)^ The Cronbach's alpha coefficient values of 0.87 and 0.88 were shown in the Spanish version;^(14)^ 0.81 and 0.87 in the German version;^(13)^ and 0.91 and 0.88 in the Portuguese version, all values for both SDI-2 subscales, respectively.^(15)^ However, additional techniques were included for improved precision of estimates,^(35)^ such as the McDonald's Omega and Greatest Lower Bound (GLB), which showed satisfactory results for instrument reliability.

As recommended by the literature,^(31)^ several complementary indexes were used to evaluate the quality of the factorial solution. The Generalized H (GH), the FDI, and the ORION marginal reliability indexes were used to ensure quality, replicability, and accuracy of the factor solution. Thus, the Generalized H (GH) allowed measuring the maximum proportion of the factor variance. The FDI allowed determining that the factor score estimates represented the latent factor scores with an adequate factorial structure. The ORION marginal reliability index allowed estimation of correlations corresponding to the same factor measured with the different models.^(31)^

The metrical invariance results indicate that the psychometric properties of the SDI-2 are maintained in both male and female populations, and do not undergo significant changes in their properties. The measurement of invariance allows examining whether an instrument has the same psychometric properties between heterogeneous groups.^(36)^

Despite the results found, the study was not without limitations, one of which was the lower participation of men than women in the study, and other was the collected sample revealed a younger mean age.

The set of techniques and indicators that were used in the SDI-2 for testing the construct in all stages—dimensionality, extraction, rotation, quality, stability, and invariance of the model—are unpublished. In addition, they made it possible to identify great data consistency and evidence of validation of the investigated instrument.

## Conclusion

The Brazilian version of the SDI-2 is a self-report, valid, reliable and invariant across sex. The SDI −2 can be used in research and clinical practice and may improve the estimated of the prevalence of HSDD in Brazilian population.
